# Prognostic value and immunological significance of t-cell proliferation regulators in pancreatic cancer: a novel predictive model

**DOI:** 10.3389/fmed.2026.1800810

**Published:** 2026-05-05

**Authors:** Wenyi Guo, Qingeng Wei, Yibo Wang, Hao Zhang, Jiacheng Zhang, Hengyu Liu, Yike Zhang, Mengyao Sun, Xuyang Ding, Lei Wang, Jianwei Xu

**Affiliations:** Department of Pancreatic Surgery, General Surgery, Qilu Hospital, Cheeloo College of Medicine, Shandong University, Jinan, China

**Keywords:** IL1RN, molecular subtypes, pancreatic cancer, prognostic model, T-cell regulation

## Abstract

**Background:**

Pancreatic cancer (PC) is characterized by a highly immunosuppressive microenvironment, limiting the efficacy of immunotherapy. T-cell regulation plays a pivotal role in anti-tumor immunity. However, the prognostic value and therapeutic implications of T-cell regulation-related genes (TRGs) in PC remain largely unexplored. This study aims to construct a TRG-based risk model and validate key therapeutic targets.

**Methods:**

Transcriptomic profiles and clinical data were retrieved from TCGA and GEO databases. Non-negative Matrix Factorization (NMF) clustering was performed to identify molecular subtypes. A prognostic risk signature was constructed using LASSO regression analysis. The immune landscape was evaluated via ssGSEA, CIBERSORT and TIDE algorithms. A nomogram was established to predict overall survival, and drug sensitivity was assessed based on IC50 estimation. Crucially, the expression of the key signature gene IL1RN was validated in clinical tissues using qRT-PCR, Western Blot, and immunohistochemistry (IHC). Its function in T-cell mediated cytotoxicity was further verified using co-culture assays.

**Results:**

Two distinct molecular subtypes were identified, with Cluster 1 exhibiting an immune-hot phenotype and superior survival. A robust risk model was constructed and validated, serving as an independent prognostic factor. High-risk patients were characterized by an immune-excluded phenotype but showed higher sensitivity to chemotherapeutic agents like Gemcitabine. Experimental validation confirmed that IL1RN was significantly upregulated in PC tissues. Functionally, knockdown of IL1RN significantly enhanced the cytotoxicity of T cells against pancreatic cancer cells *in vitro*.

**Conclusion:**

We constructed a novel TRG-based prognostic signature that can serve as an auxiliary tool for clinical prognostic evaluation in pancreatic cancer. Furthermore, we validated IL1RN as a critical mechanism of immune resistance. IL1RN is expected to become a novel target for pancreatic cancer immunotherapy, providing experimental evidence for the stratified treatment of PC.

## 1 Introduction

Pancreatic cancer (PC) is recognized as one of the most lethal malignancies, ranking seventh globally and sixth in China among cancer-related mortality causes (1–3). Characterized by its aggressive nature and rapid disease progression, PC is notorious for its poor prognosis, with a dismal 5-year overall survival rate of merely 10% (4, 5). While surgical resection remains the most effective treatment for patients diagnosed at an early stage, advancements in chemotherapy and immunotherapy have yielded limited improvements in patient outcomes, primarily due to the unique tumor microenvironment characterized by hypoxia and metabolic reprograming (6–12). Recently, breakthroughs in targeted therapies, particularly KRAS G12D inhibitors (such as MRTX1133), and personalized immunotherapeutic strategies, notably mRNA neoantigen vaccines, have demonstrated preliminary but promising efficacy in overcoming these highly immunosuppressive barrier (13, 14). This underscores the urgent need to explore novel biomarkers for assessing the prognosis of PC patients to extend their survival (15). Although previous studies have identified individual factors contributing to T cell exhaustion (16–18), there remains a significant gap in the systematic analysis of T cell proliferation-related gene signatures, which could unveil new biomarkers and therapeutic targets (19–21). This study aims to define a comprehensive gene signature associated with T cell proliferation regulation, utilizing data from The Cancer Genome Atlas (TCGA) (22) to investigate the molecular interactions between T cell receptor signaling (TCRS) components and tumor progression (23, 24). The findings indicate that dysregulation of T cell proliferation genes correlates with poorer survival outcomes, establishing TCRS as a critical determinant of immune evasion in PC and providing a framework for patient stratification in personalized immunomodulatory therapies (25–28).

Our study focuses on the phenotypic characteristics of T cell proliferation and its regulatory mechanisms in the context of PC. Previous research has highlighted the critical role of T cell dysfunction in promoting immune evasion (16, 21), yet the specific contributions of T cell proliferation-related genes remain inadequately explored. Our investigation reveals a significant association between the dysregulation of T cell proliferation genes and poor survival outcomes in PC patients, underscoring the potential of these genes as novel biomarkers for prognostic assessment. By leveraging the comprehensive data from TCGA, we elucidate the molecular interactions between TCRS components and tumor progression, establishing TCRS as a pivotal determinant of immune evasion in PC (23). The advantage of this approach lies in its ability to systematically characterize the gene expression profiles associated with T cell proliferation regulators, which are critical in understanding the immune evasion mechanisms inherent to PC (18, 20). This novel insight not only enhances our understanding of the tumor microenvironment but also lays the groundwork for personalized immunomodulatory therapies aimed at improving patient stratification and outcomes (27, 28).

The primary objective of this research is to delineate a robust gene signature related to T cell proliferation modulation, thereby identifying potential biomarkers and therapeutic targets that could enhance patient stratification for personalized immunotherapeutic interventions. This study elucidates the dysregulation of T cell proliferation genes and their correlation with survival outcomes, aiming to contribute significantly to the ongoing efforts in improving prognostic assessments and treatment strategies for patients afflicted with this highly aggressive malignancy.

## 2 Materials and methods

### 2.1 Data acquisition and processing

Publicly available gene expression data and corresponding clinical annotations for PC were retrospectively collected from The Cancer Genome Atlas (TCGA) database.^1^ Patients lacking complete overall survival information (e.g., follow-up duration ≤ 30 days) or with insufficient clinical data (e.g., unknown age or tumor stage) were excluded, resulting in a training cohort of 182 samples (178 PC tissues and 4 adjacent normal tissues). For external validation, an independent cohort GSE183795 consisting of 134 PC tumor tissues was obtained from the Gene Expression Omnibus (GEO) database. The detailed baseline clinicopathological characteristics of the included patients are summarized in Table 1. A list of 35 TRGs was retrieved from previous studies (29). The genetic alteration landscape, including Copy Number Variations (CNV), was analyzed and visualized using data from the TCGA database.

**TABLE 1:** The detailed baseline clinicopathological characteristics of the included patients.

Characteristics	TCGA cohort (*N* = 178)
Age (years)	
<65	77 (43.3%)
≥65	101 (56.7%)
Gender	
Male	98 (55.1%)
Female	80 (44.9%)
Tumor stage (AJCC)	
Stage I–II	166 (93.3%)
Stage III–IV	7 (3.9%)
Unknown	5 (2.8%)
Histological grade	
G1-G2 (well/moderate)	123 (69.1%)
G3-G4 (poor/undifferentiated)	52 (29.2%)
Unknown	3 (1.7%)

### 2.2 Identification of differentially expressed TRGs and functional enrichment

To explore the biological functions and signaling pathways associated with these genes, Gene Ontology (GO) annotation and Kyoto Encyclopedia of Genes and Genomes (KEGG) pathway enrichment analyses were conducted using the “clusterProfiler” R package (30).

### 2.3 Non-negative matrix factorization (NMF) clustering

To identify distinct molecular subtypes, univariate Cox regression analysis was first applied to screen for TRGs with prognostic value (*P* < 0.05). Based on the expression profiles of these prognostic genes, clustering was performed using the Non-negative Matrix Factorization (NMF) algorithm via the “NMF” R package. The standard “brunet” method was utilized with 50 iterations. The optimal number of clusters (k) was determined based on the cophenetic correlation coefficient, dispersion, and silhouette scores (Supplementary Figure 1). The heterogeneity between the identified clusters was further verified using UMAP. Gene Set Variation Analysis (GSVA) was performed to investigate the differences in biological pathway activity between subtypes. The heterogeneity between the identified clusters was further verified using Uniform Manifold Approximation and Projection (UMAP) for dimensionality reduction.

### 2.4 Immune infiltration and microenvironment characterization

To comprehensively evaluate the immune landscape of the tumor microenvironment, two distinct algorithms were employed. First, the CIBERSORT algorithm (31) was applied to estimate the relative proportions of 22 human immune cell types based on the LM22 signature matrix. The results were visualized as box plots and stacked bar charts to display the cellular composition. Second, to complement these findings, the single-sample Gene Set Enrichment Analysis (ssGSEA) algorithm was utilized via the “GSVA” R package [36.37] to calculate the infiltration scores of 28 immune cell types. Furthermore, the expression levels of immune checkpoints (e.g., PDCD1, CTLA4), chemokines, and chemokine receptors were compared between the molecular subtypes. Statistical differences were determined using the Wilcoxon rank-sum test.

### 2.5 Construction and validation of the prognostic risk model

The prognostic model was constructed using the TCGA cohort as the training set. Prior to downstream modeling, the raw gene expression profiles were normalized to log_2_(TPM + 1) format to ensure data comparability and model reproducibility. Least Absolute Shrinkage and Selection Operator (LASSO) regression analysis was performed using the “glmnet” R package to minimize overfitting and identify the most robust prognostic genes. The optimal penalty parameter (λ) was determined via 10-fold cross-validation. The final risk signature was strictly constructed based on the candidate genes that retained non-zero regression coefficients at the optimal λ value (λ = 0.0498). Based on the optimal λ, a risk score was calculated for each patient using the following formula:


$$Risk\;Score=\overset{\text{n}}{\underset{\text{i}=1}{\sum }}\left(C\,{\text{oef}}_{\text{i}}\,\times {\text{Exp}}_{\text{i}}\right)$$

Coef_*i*_ represents the regression coefficient of the i-th gene derived from the LASSO analysis, and Exp_*i*_ denotes the expression level of that gene. Patients were categorized into high- and low-risk groups based on the median risk score. Kaplan-Meier survival analysis and time-dependent Receiver Operating Characteristic (ROC) curves were generated to evaluate the model’s predictive performance, which was subsequently validated in the external cohort.

### 2.6 Establishment of a predictive nomogram

Univariate and multivariate Cox regression analyses were conducted to determine the independent prognostic value of the risk score and other clinicopathological features (e.g., age, tumor stage, grade). Based on the identified independent predictors, a nomogram was constructed using the “rms” R package (32) to predict the 1-, 2-, and 3-year overall survival probability. The performance of the nomogram was assessed using calibration curves to evaluate the consistency between predicted and observed survival, and Decision Curve Analysis (DCA) to evaluate the net clinical benefit.

### 2.7 Prediction of therapeutic response

To assess the potential response to immune checkpoint blockade (ICB), the Tumor Immune Dysfunction and Exclusion (TIDE) algorithm^2^ was employed (33). TIDE scores, along with T-cell dysfunction and exclusion scores, were calculated, where a higher TIDE score indicates a higher potential for immune evasion. Drug sensitivity prediction for common chemotherapeutic agents was performed using the “pRRophetic” R package (34). The half-maximal inhibitory concentration (IC50) was estimated based on the Genomics of Drug Sensitivity in Cancer (GDSC) database. Lower IC50 values represent higher drug sensitivity.

### 2.8 Human tissue samples and ethics

Clinical data and tissue specimens were collected from patients undergoing surgery at the Department of Pancreatic Surgery, Qilu Hospital of Shandong University. Paraffin-embedded PC tissue arrays representing six patients were manufactured by Chengdu Lilai Biomedicine (Chengdu, China). The study protocol received ethical approval from the Institutional Review Board of Qilu Hospital (Approval No. KLYY-202402-009-1). All patients signed written informed consent forms at the time of inclusion. The detailed clinicopathological characteristics of these 6 patients, including age, sex, tumor stage, and differentiation grade, are summarized in Table 2.

**TABLE 2:** The detailed clinicopathological characteristics of the 6 patients.

Patient ID	Age (years)	Gender	Tumor location	TNM stage	Differentiation grade
Patient 1	65	Male	Head	IIA	Moderate
Patient 2	68	Male	Head	IIA	Well
Patient 3	72	Female	Body	III	Poor
Patient 4	64	Male	Tail	III	Poor
Patient 5	70	Male	Tail	IIB	Moderate
Patient 6	69	Female	Head	III	Poor

### 2.9 Histology and IHC analysis

Tissue samples were fixed with 4% paraformaldehyde, paraffin-embedded, and cut into 4-μm sections. For IHC assay, tissues were stained for interested protein using a streptavidin-biotin peroxidase method. The primary antibody was used as follows: IL1RN (83335-4-RR, Proteintech). IHC staining was scored based on staining intensity (0: negative; 1: weak, light brown; 2: moderate, brown; 3: strong, dark brown) and the percentage of positive cells. For each sample, 5–10 random high-power fields (× 400) were examined. The H-score was determined by the formula: H-score = (%weak × 1) + (%moderate × 2) + (%strong × 3). The final scores ranged from 0 to 300, directly correlating with antigen expression levels.

### 2.10 Mice CD8 + T cells isolation

Mice CD8 + T cells were isolated from C57BL/6J mice spleens using a CD8a + T Cell Isolation Kit (130-104-075; Miltenyi Biotec), and a LS Column (130-042-401; Miltenyi Biotec), and a MidiMACS™ Separator (130-042-301; Miltenyi Biotec). CD8 + T cells were stimulated with CD3/CD28 monoclonal antibodies (CF0019, CF0029, NeoMab) and recombinant Murine interleukin 2 (IL-2) (CM003, Chamot) in Lymphocyte serum-free Medium KBM 581 (88581CM059, Corning) for 3 days.

### 2.11 Transduction of CD8 + T cell

To knockdown IL1RN expression, lentiviral vectors encoding short hairpin RNA (shRNA) targeting IL1RN (sh-IL1RN-1, 5’ GGTACCCATTGAGCCTCATGC 3’; sh-IL1RN-2, 5’ GCCCGTCAGCCTCACCAATAT 3’; sh-IL1RN-3, 5’ GCCTGTTCCCATTCTTGCATG 3’) and a non-targeting scramble control (sh-NC, 5’ TTCTCCGAACGTGTCACGT 3’) were synthesized by GenePharma (Shanghai, China).

The activated CD8 + T cells were then transduced with the lentiviral particles at a multiplicity of infection (MOI) of 10 and a lentiviral titer of 5*10^8^ TU/mL in the presence of 4 μg/mL polybrene. After 24 h of incubation, the culture medium was replaced with fresh complete medium containing IL-2. The knockdown efficiency of IL1RN was subsequently verified by Western blot.

### 2.12 Cell lines and cell culture

The murine pancreatic cancer cell lines Panc02 and KPC were used in this study. Both cell lines were cultured in Dulbecco’s Modified Eagle Medium (DMEM, Gibco) supplemented with 10% fetal bovine serum (FBS; Gibco) and 1% penicillin-streptomycin. Cells were maintained in a humidified incubator at 37°C with 5% CO_2_.

### 2.13 T cell-mediated tumor cell killing assay

The activated T cells transfected with lentiviral vectors were allowed to adhere to the plates overnight and then co-cultured with tumor cells for 48 h at a ratio of 1:10. T cells and cell debris were removed and washed three times using PBS. The remaining living cancer cells were fixed with 4% paraformaldehyde, stained with 0.5% crystal violet, and OD at 570 nm was quantified using a spectrophotometer. All co-culture and cell-killing assays were performed with five independent biological replicates (*n* = 5).

### 2.14 Western blot analysis

After extracting from pancreatic cancer cell/tissues, total proteins were separated on 10% SDS-PAGE gel and transferred to polyvinylidene difluoride (PVDF) membrane. The membranes were blocked in 5% skim milk for 2 h and then incubated with primary antibodies at 4 °C overnight. Then membranes were incubated with fluorescently conjugated secondary antibodies (SA00001-2, Proteintech, Wuhan, China). Immuno-reactive complexes were visualized using NcmECL High (NCM Biotech). Primary antibodies were used as follows: IL1RN (83335-4-RR, Proteintech); GAPDH (60001-4-lg, Proteintech).

### 2.15 The quantitative reverse transcription-polymerase chain reaction (qRT-PCR) assay

Total RNA was isolated from paired pancreatic tumor and matched adjacent non-tumor tissues using FastPure Cell/Tissue Total RNA Isolation Kit (Vazyme, Nanjing China). Subsequently, the HiScript II Q RT SuperMix kit (Vazyme) was used to transcribe total RNA as cDNA. Amplified products were detected with SYBR Green (Vazyme). GAPDH was selected as the endogenetic references. The specific primers contained IL1RN: 5’-CTTCTATCTGAGGAACAACCAACTA-3’(forward, F), 5’-ACACAGGACAGGCACATCTT-3’ (reverse, R).

### 2.16 Statistical analysis

All statistical analyses were performed using R software (version 4.4.1). Continuous variables were compared using the Wilcoxon rank-sum test or Student’s *t*-test. Categorical variables were compared using the Chi-square test. Survival differences were analyzed using the log-rank test. A two-sided *P*-value < 0.05 was considered statistically significant.

## 3 Results

### 3.1 Landscape of genetic variation and expression of T-cell regulation-related genes in pancreatic cancer

**FIGURE 1:**
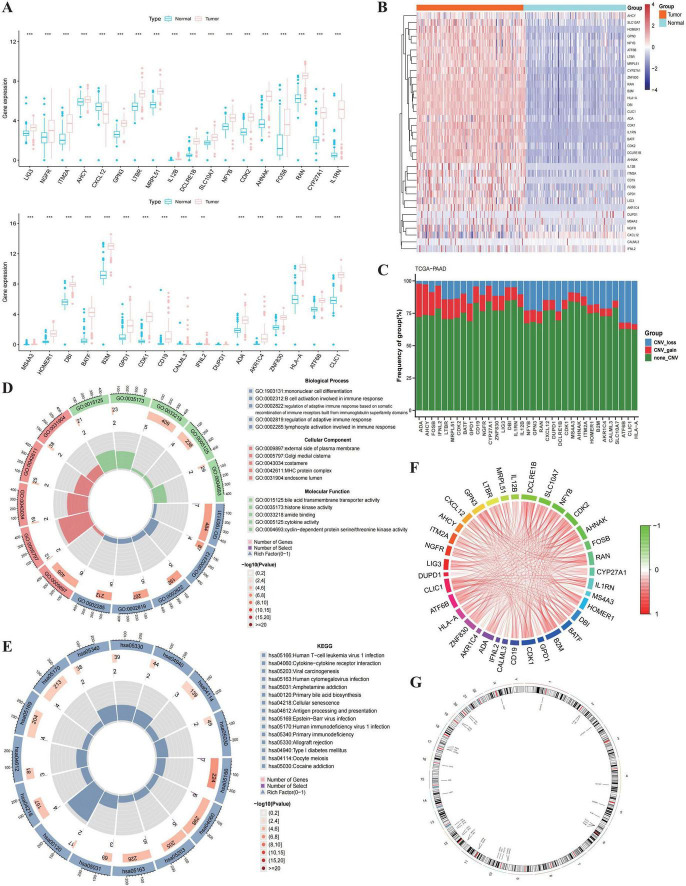
The expression and genomic features of T cell proliferation regulators in PC. **(A)** The differential expression of T cell proliferation regulators between PC and normal samples. **(B)** The heatmap of differentially expressed genes. In the middle annotation bar, non-cancerous and PC samples are indicated in blue and red colors, respectively. The intensity of the color in the heatmap signifies gene expression density per sample, with darker colors indicating higher density. The y-axis of the lower heatmap represents genes (red and blue colors indicate upregulation and downregulation, respectively). **(C)** The CNV mutation frequency of 35 T cell proliferation regulators. **(D)** Gene Ontology (GO) enrichment (Top 5). Each column represents a GO term. The color of the column represents different GO categories. The length represents the number of genes enriched in the term. **(E)** Kyoto Encyclopedia of Genes and Genomes (KEGG) enrichment. The color transition from red (high) to blue (low) indicates log2 (FC) values of the genes. Each pathway is represented by a distinct color. **(F)** Correlation String Diagram of T Cell Proliferation Regulators. **(G)** Chromosome position and alteration of T cell proliferation regulators. Statistical methods: Differential expression was evaluated using the Wilcoxon rank-sum test. *p < 0.05, **p < 0.01, ***p < 0.001.

To systematically identify TRGs involved in the progression of PC, we first evaluated the expression patterns of candidate genes in the TCGA-PAAD cohort. As illustrated in Figure 1A, most TRGs exhibited significant dysregulation between tumor and normal tissues. Consistent with this, the heatmap visualization confirmed distinct expression profiles, effectively distinguishing PC samples from normal controls (Figure 1B).

We further investigated the genetic alterations of these TRGs to understand the potential mechanisms driving their aberrant expression. The analysis of CNV revealed widespread genomic frequency alterations among the identified genes. As shown in Figure 1C, genes such as AHNAK and CDK2 showed frequent CNV gain, while others exhibited CNV loss, suggesting that genomic instability contributes to the dysregulation of T-cell regulation-related regulators in PC.

To explore the biological functions and signaling pathways associated with these genes, we performed functional enrichment analyses. GO annotation (Figure 1D) demonstrated that these genes were primarily enriched in immune-related biological processes, including “mononuclear cell differentiation,” “regulation of adaptive immune response,” and “T cell activation.” Similarly, KEGG pathway analysis (Figure 1E) highlighted the enrichment of pathways critical for immune modulation.

Furthermore, the correlation network (Figure 1F) and chromosomal distribution (Figure 1G) of these genes provided a comprehensive view of their intracellular interactions and genomic locations, indicating a complex regulatory network governing T-cell function within the pancreatic tumor microenvironment.

### 3.2 Identification of molecular subtypes based on T-cell proliferation regulators and their association with prognosis in pancreatic cancer

**FIGURE 2:**
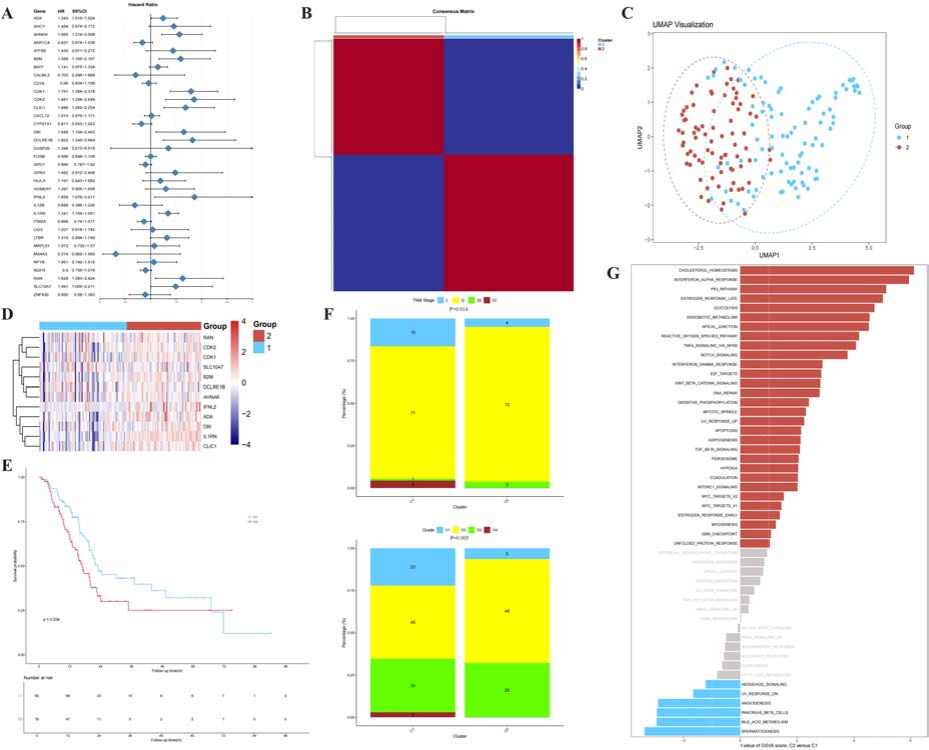
Identification and prognostic validation of T-cell regulation-related molecular subtypes. **(A)** Forest plot of univariate Cox regression analysis showing the Hazard Ratios (HR) and 95% Confidence Intervals (CI) of prognostic TRGs. **(B)** Consensus clustering matrix of PC samples for *k* = 2, showing two distinct clusters. **(C)** UMAP visualization confirming the spatial distribution of the two molecular subtypes. **(D)** Heatmap displaying the differential expression of TRGs between the two identified clusters. **(E)** Kaplan-Meier survival curves showing the Overall Survival (OS) difference between Cluster 1 and Cluster 2 (*P* = 0.034). **(F)** Comparison of clinical characteristics, including Stage and Grade, between the two clusters (*P* = 0.005 for Grade). **(G)** GSVA analysis showing the activation of biological pathways in the two subtypes. The bar length represents the *t*-value of the GSVA score. Statistical methods: Survival differences were assessed using the log-rank test. Group comparisons for clinical data were performed using the Chi-square test or Wilcoxon rank-sum test.

To explore the heterogeneity of T-cell regulation patterns in PC, we first performed univariate Cox regression analysis to screen for genes with prognostic value. As shown in Figure 2A, TRGs were significantly associated with overall survival. Based on the expression profiles of these prognostic genes, we applied the NMF algorithm to classify the PC patients. The optimal number of clusters (*k* = 2) was determined based on the cophenetic correlation coefficient, dispersion, and the consistency of the consensus map (Figure 2B), identifying two distinct molecular subtypes (Cluster 1 and Cluster 2). The distinction between the two subtypes was further validated by UMAP visualization (Figure 2C) and a differential expression heatmap (Figure 2D).

To evaluate the clinical relevance of these subtypes, we performed Kaplan-Meier survival analysis. As illustrated in Figure 2E, patients in Cluster 1 exhibited significantly better overall survival (OS) compared to those in Cluster 2 (*P* = 0.034). Furthermore, we analyzed the distribution of clinicopathological features between the two subtypes. As shown in Figure 2F, Cluster 2 was characterized by a significantly higher proportion of patients with advanced TNM stage (*P* = 0.014) and higher histological grade (*P* = 0.005), which is consistent with its poorer prognosis and more aggressive clinical phenotype.

To uncover the biological mechanisms underlying these subtypes, we performed GSVA enrichment analysis (Figure 2G). The results revealed that Cluster 1 was markedly enriched in immune-activation pathways. In contrast, Cluster 2 showed enrichment in stromal and oncogenic pathways, indicating an immunosuppressive and metabolically active tumor microenvironment.

### 3.3 Distinct immune infiltration landscapes and immunomodulatory profiles of the molecular subtypes

**FIGURE 3:**
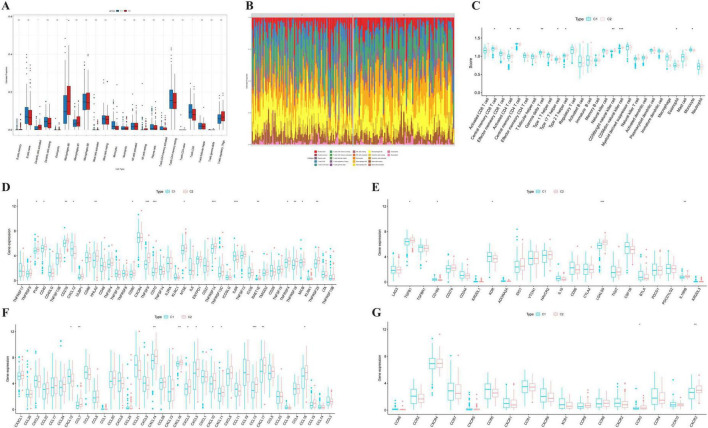
Comparison of immune infiltration and immunomodulatory molecules between the two molecular subtypes. **(A)** Box plots showing the differences in immune-related scores between Cluster 1 and Cluster 2. **(B)** Stacked bar chart illustrating the relative abundance of infiltrating immune cells in each sample. **(C)** Box plots comparing the infiltration levels of 28 types of immune cells between the two clusters. **(D)** Differential expression of immunomodulator genes between Cluster 1 and Cluster 2. **(E)** Comparison of immune checkpoint gene expression (e.g., PDCD1, CTLA4, LAG3) between the two subtypes. **(G)** Differential expression of chemokines **(F)** and chemokine receptors **(G)** between the two clusters. Statistical methods: Differences between the two molecular subtypes were evaluated using the Wilcoxon rank-sum test. *p < 0.05, **p < 0.01, ***p < 0.001.

To gain deeper insights into the immunological discrepancies between the two subtypes, we evaluated the immune cell infiltration landscape using the ssGSEA and CIBERSORT algorithm. As shown in Figures 3A,B, the global immune patterns varied between the two groups. Specifically, the analysis of immune cell abundance (Figure 3C) identified significant differences in specific innate and adaptive immune cell subsets, including Natural Killer (NK) cells and T cell subpopulations, indicating a distinct immune composition in the tumor microenvironment.

Subsequently, we investigated the expression of immune-related molecules to understand the regulatory mechanisms. The analysis of immunomodulators (Figure 3D) and immune checkpoints (Figure 3E) revealed that Cluster 1 exhibited significant differential expression of key immune-regulatory genes compared to Cluster 2. Notably, genes associated with immune co-stimulation and coinhibition, such as LGALS9, showed distinct expression patterns, suggesting potential differences in immune response regulation.

In addition, to elucidate the mechanisms of immune cell recruitment, we analyzed the expression of chemokines and their receptors. As illustrated in Figures 3F,G, several key chemokines and receptors, including CXCR3, were differentially expressed between the two clusters. These results suggest that the two molecular subtypes possess fundamentally different chemokine milieus, which may drive the observed variations in immune cell infiltration.

### 3.4 Construction and validation of a T-cell regulation-related prognostic risk model

**FIGURE 4:**
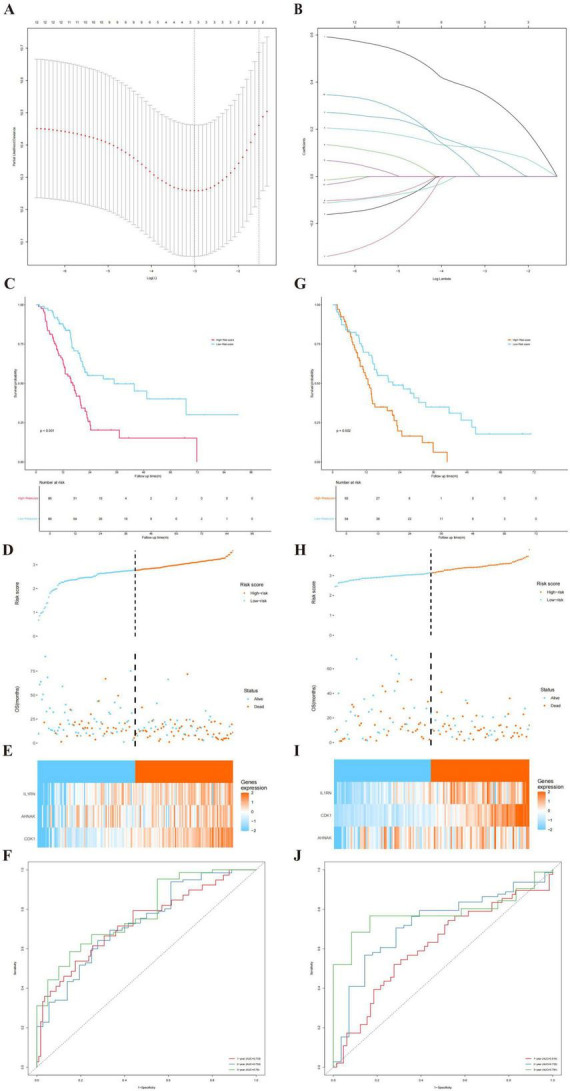
Construction and validation of the T-cell regulation-related prognostic risk model. (A-B) LASSO Cox regression analysis. **(A)** Tuning parameter (λ) selection using cross-validation. The vertical dotted lines indicate the optimal λ values based on the minimum criteria and the 1-standard error of the minimum criteria. **(B)** LASSO coefficient profiles of the candidate genes. Each curve represents a single gene. The three genes (CDK1, AHNAK, and IL1RN) with non-zero coefficients at the optimal λ were selected. **(C–F)** Validation of the risk model in the [Training/TCGA] cohort. **(C)** Kaplan-Meier survival curve comparing high- and low-risk groups. **(D)** Distribution of risk scores and survival status. **(E)** Heatmap showing the expression of signature genes in high- and low-risk groups. **(F)** Time-dependent ROC curves for predicting 1-, 3-, and 5-year overall survival (AUC = 0.733, 0.732, and 0.780, respectively). **(G–J)** Validation of the risk model in the [Validation/GEO] cohort. **(G)** Kaplan-Meier survival analysis. **(H)** Risk score distribution and survival status. **(I)** Expression heatmap of signature genes. **(J)** Time-dependent ROC curves. (AUC = 0.614, 0.723, and 0.781, respectively). Patients were divided into high-risk and low-risk groups based on the median risk score. Statistical methods: Survival differences were compared via the log-rank test.

To quantify the prognostic value of T-cell regulation patterns in individual patients, we constructed a risk scoring model using LASSO regression analysis. As illustrated in Figures 4A,B, the optimal penalty parameter (λ) was determined through cross-validation to prevent overfitting. By applying this optimal λ value, three core genes (CDK1, AHNAK, and IL1RN) with non-zero regression coefficients were strictly selected from the candidate list to construct a robust prognostic signature. Based on the coefficients derived from this analysis, a risk score was calculated for each patient as follows: Risk Score = (0.348 × Expression of CDK1) + (0.097 × Expression of AHNAK) + (0.119 × Expression of IL1RN). We first evaluated the model’s performance in the TCGA cohort. Patients were stratified into high- and low-risk groups based on the median risk score. Kaplan-Meier analysis indicated that patients in the high-risk group had a significantly poorer OS compared to those in the low-risk group (Figure 4C, *P* < 0.001). The distribution of risk scores and survival status (Figure 4D) further demonstrated that mortality rates increased with rising risk scores. Additionally, the heatmap (Figure 4E) revealed distinct expression patterns of the signature genes between the two groups. The predictive accuracy of the model was assessed using time-dependent ROC curves (Figure 4F), which showed satisfactory Area Under the Curve (AUC) values for 1-, 3-, and 5-year survival, indicating high sensitivity and specificity (AUC = 0.733, 0.732, and 0.780, respectively).

Subsequently, the robustness of the risk model was verified in an independent GEO cohort. Consistent with the training set, the high-risk group in the validation cohort exhibited significantly reduced survival time (Figure 4G, *P* = 0.002). The risk score distribution (Figure 4H) and gene expression profiles (Figure 4I) aligned well with the initial findings. Moreover, the ROC analysis (Figure 4J) confirmed the model’s stable prognostic ability across different datasets (AUC = 0.614, 0.723, and 0.781, respectively).

### 3.5 Establishment and evaluation of a predictive nomogram

**FIGURE 5:**
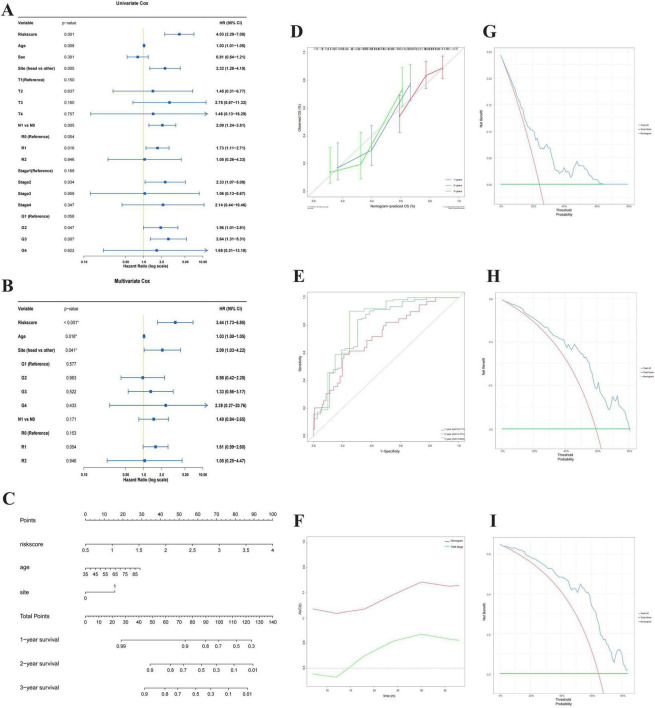
Construction and evaluation of a nomogram for predicting overall survival. **(A,B)** Forest plots of univariate **(A)** and multivariate **(B)** Cox regression analyses identifying independent prognostic factors. **(B,C)** A nomogram for predicting 1-, 2-, and 3-year overall survival rates of PC patients, incorporating the risk score, age, and tumor site. **(D)** Calibration curves assessing the agreement between nomogram-predicted and observed survival probabilities at 1, 2, and 3 years. **(E)** ROC curves comparing the predictive sensitivity and specificity of the nomogram against other clinical features. **(F)** Time-dependent AUC plot showing the predictive accuracy of the model over time. **(G–I)** Decision Curve Analysis (DCA) evaluating the clinical net benefit of the nomogram for 1-year **(G)**, 2-year **(H)**, and 3-year **(I)** survival prediction. Statistical methods: Wald test was used for Cox regression. Calibration was assessed using the bootstrap method (1,000 replicates). The predictive accuracy was quantified by the C-index.

To determine whether the risk score could serve as an independent prognostic factor, we performed univariate and multivariate Cox regression analyses including clinical characteristics. Univariate analysis (Figure 5A) indicated that the risk score (HR = 4.03, 95% CI: 2.29–7.08, *P* = 0.001), age (HR = 1.03, 95% CI: 1.01–1.05, *P* = 0.003), tumor site (HR = 2.32, 95% CI: 1.28–4.19, *P* = 0.006), N stage (HR = 2.09, 95% CI: 1.24–3.51, *P* = 0.005) and tumor grade were significantly associated with overall survival. Importantly, multivariate analysis (Figure 5B) confirmed that the risk score remained a robust independent prognostic indicator (HR = 3.44, 95% CI: 1.73–6.85, *P* < 0.001) after adjusting for confounding factors. Additionally, Age (HR = 1.03, 95% CI: 1.00–1.05, *P* = 0.018) and Tumor Site (HR = 2.09, 95% CI: 1.03–4.22, *P* = 0.041) were also identified as independent predictors for PC patients.

Based on these independent prognostic factors (Risk score, Age, and Site), we constructed a nomogram (Figure 5C) to quantitatively predict the 1-, 2-, and 3-year OS probability for PC patients. The nomogram achieved a high C-index of 0.772 (95% CI: 0.725–0.819). To validate the predictive performance of the nomogram, we employed calibration curves (Figure 5D), which demonstrated excellent agreement between the predicted and observed survival probabilities at 1, 2, and 3 years.

Furthermore, the predictive accuracy was evaluated using ROC analysis (Figure 5E), where the nomogram exhibited a superior Area Under the Curve (AUC). The time-dependent AUC plot (Figure 5F) further confirmed the stable and robust predictive ability of the nomogram over time. Finally, Decision Curve Analysis (DCA) for 1-, 2-, and 3-year survival (Figures 5G–I) demonstrated that the nomogram yielded a higher net benefit within a reasonable range of threshold probabilities, highlighting its potential clinical utility for personalized decision-making.

### 3.6 Prediction of immunotherapy response and chemotherapy sensitivity

**FIGURE 6:**
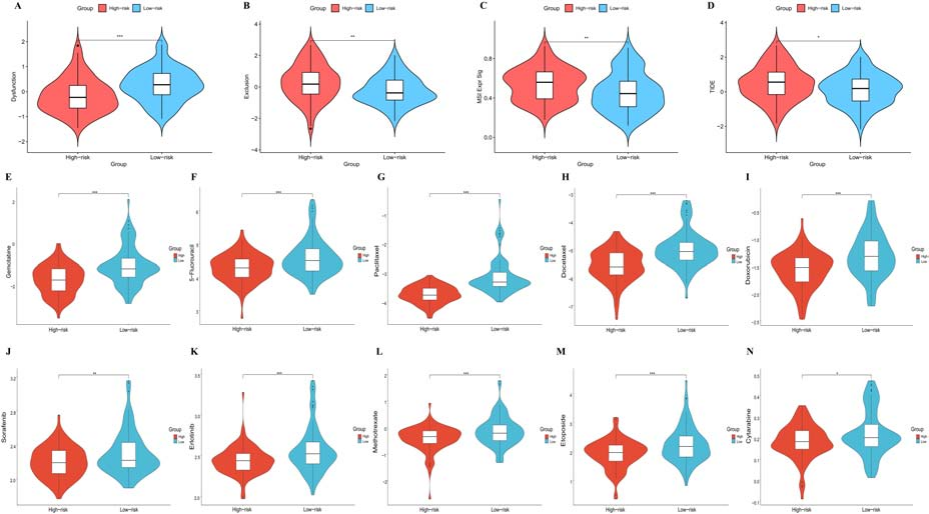
Prediction of therapeutic response to immunotherapy and chemotherapy in high- and low-risk groups. **(A–D)** Evaluation of immune response potential using the TIDE algorithm. Violin plots illustrating the significant differences in **(A)** T-cell Dysfunction score (*P* < 0.001), **(B)** T-cell Exclusion score (*P* < 0.01), **(C)** MSI expression signature (*P* < 0.01), and **(D)** overall TIDE score (*P* < 0.05) between the two risk groups. **(E–N)** Comparison of estimated drug sensitivity (IC50 values) between high- and low-risk groups. The drugs analyzed include Gemcitabine **(E)**, 5-Fluorouracil **(F)**, Paclitaxel **(G)**, Docetaxel **(H)**, Doxorubicin **(I)**, Sorafenib **(J)**, Erlotinib **(K)**, Methotrexate **(L)**, Etoposide **(M)**, and Cytarabine **(N)**. A lower IC50 value indicates higher drug sensitivity. Risk groups were defined by the median risk score. Statistical methods: Differences between the two risk groups were assessed using the Wilcoxon rank-sum test. *p < 0.05, **p < 0.01, ***p < 0.001.

To explore personalized therapeutic strategies, we evaluated the potential response to immune checkpoint blockade (ICB) using the TIDE (Tumor Immune Dysfunction and Exclusion) algorithm. The overall TIDE score, a robust predictor of immune evasion, was significantly higher in the high-risk group compared to the low-risk group (Figure 6D, *P* < 0.05), indicating a lower likelihood of benefiting from ICB therapy.

To decipher the underlying mechanisms of this resistance, we further analyzed the specific TIDE components. Interestingly, the high-risk group exhibited a significantly lower T-cell Dysfunction score (Figure 6A, *P* < 0.001) but a markedly elevated T-cell Exclusion score (Figure 6B, *P* < 0.01). This discrepancy suggests that the immune resistance in high-risk patients is primarily driven by the “exclusion” phenotype—where effector T cells are prevented from infiltrating the tumor microenvironment—rather than by T-cell exhaustion or dysfunction itself. Additionally, the Microsatellite Instability (MSI) expression signature was found to be significantly higher in the high-risk group (Figure 6C, *P* < 0.01), reflecting distinct genomic instability patterns associated with the risk signature.

Given the limited efficacy of immunotherapy for high-risk patients, we further investigated their susceptibility to common chemotherapeutic and targeted drugs by estimating the half-maximal inhibitory concentration (IC50). Notably, the analysis revealed that the high-risk group had significantly lower estimated IC50 values for several first-line pancreatic cancer drugs, including Gemcitabine (Figure 6E) and 5-Fluorouracil (Figure 6F). Furthermore, this group also demonstrated higher sensitivity to a broader spectrum of other therapeutic agents, including Paclitaxel (Figure 6G), Docetaxel (Figure 6H), Doxorubicin (Figure 6I), Sorafenib (Figure 6J), Erlotinib (Figure 6K), Methotrexate (Figure 6L), Etoposide (Figure 6M), and Cytarabine (Figure 6N).

These findings suggest that while high-risk patients may face resistance to immunotherapy, they might benefit more from specific chemotherapy or targeted therapy regimens.

### 3.7 Experimental validation of the role of IL1RN in PC.

**FIGURE 7:**
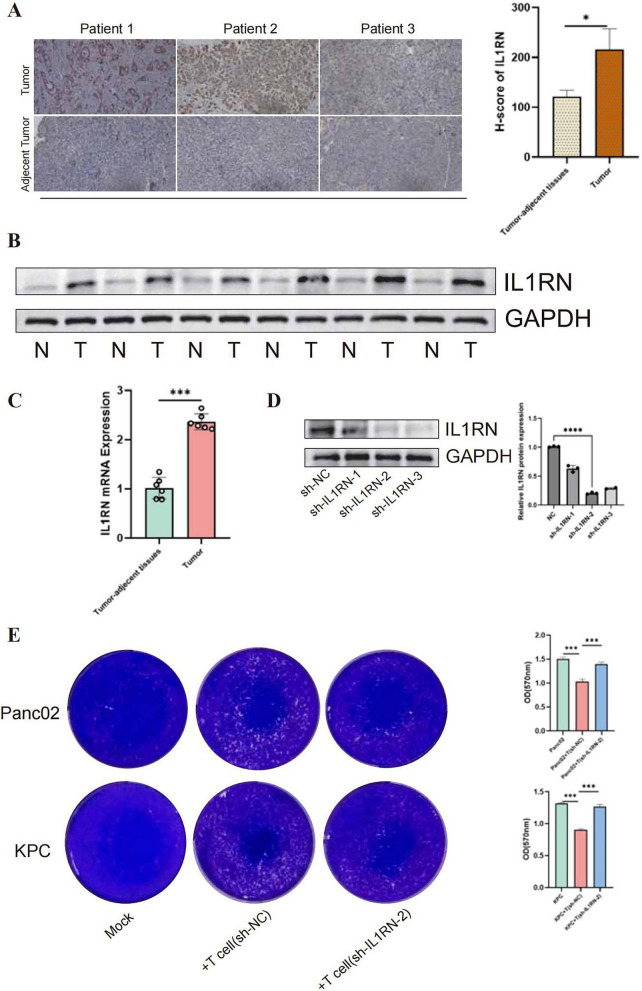
Experimental validation of the role of IL1RN in PC. **(A)** Representative immunohistochemistry (IHC) images of IL1RN in paired tumor and adjacent normal tissues from three patients. The histogram (right) shows the H-score of IL1RN staining, indicating significantly higher expression in tumor tissues (*P < 0.05). **(B)** Western blotting analysis of IL1RN expression levels in 6 paired PC tissues (T) and adjacent normal pancreatic tissue (N). **(C)** qPCR analysis of IL1RN mRNA expression levels in 6 paired PC tissues and adjacent normal pancreatic tissue. **(D)** Immunoblot of IL1RN in control and IL1RN-knockdown T cells. The accompanying bar chart displays the relative grayscale quantification of IL1RN protein expression normalized to GAPDH (*n* = 3). **(E)** Representative images (left) and statistical results (right) of T cell-mediated cancer cell-killing assay results (*n* = 5 independent biological replicates). Statistical methods: Paired data (T vs. N) were analyzed using the paired Student’s *t*-test. Multiple group comparisons were performed using One-way ANOVA followed by Dunnett’s *post-hoc* test. Data are Mean ± SD. *p < 0.05, ***p < 0.001, ****p < 0.0001.

To validate the clinical relevance and biological function of IL1RN in PC, we first examined its expression levels in clinical samples. IHC staining revealed that IL1RN protein was strongly expressed in tumor tissues, whereas it remained largely negative in adjacent normal pancreatic tissues (Figure 7A). This finding was further corroborated by Western blotting and qPCR analysis of six paired clinical samples, both of which demonstrated a significant upregulation of IL1RN at the protein and mRNA levels in tumor tissues compared to their normal counterparts (Figures 7B,C). To investigate the impact of IL1RN on the anti-tumor immune response, we utilized shRNA to knockdown IL1RN in T cells. Grayscale quantification of the Western blot bands confirmed that sh-IL1RN-2 exhibited the most significant inhibitory effect, successfully reducing IL1RN protein expression by approximately 80% compared to the sh-NC control group (Figure 7D *P*<0.0001). In T cell-mediated cancer cell-killing assays, co-culturing these T cells with Panc02 and KPC demonstrated that while T cells generally reduced cancer cell viability, the knockdown of IL1RN in T cells significantly enhanced their cytotoxic efficacy (Figure 7E). Statistical analysis of OD values (570nm) confirmed that the survival of both Panc02 and KPC cells was markedly decreased when subjected to IL1RN-deficient T cells compared to the control group. Taken together, these results suggest that IL1RN is highly expressed in PC and serves as a negative regulator of T cell-mediated anti-tumor immunity.

## 4 Discussion

PC remains one of the most lethal malignancies, largely due to its insidious onset and resistance to conventional therapies. T-cell exhaustion and dysfunction are hallmarks of the PC microenvironment, yet few prognostic tools specifically focus on the regulators of T-cell proliferation. In this study, we addressed this gap by comprehensively investigating the prognostic value and immunological significance of T-cell proliferation regulators in PC. By integrating multi-omics data from the TCGA and GEO databases, we successfully constructed and validated a novel three-gene prognostic risk model based on IL1RN, CDK1, and AHNAK. This model demonstrated robust survival prediction capabilities across both the training and validation cohorts, confirming its reliability as a stratification tool for PC patients. Previous studies have similarly highlighted the utility of multi-gene signatures in predicting PC outcomes, such as the classic six-gene signature identified by Stratford et al. (35). However, our model distinguishes itself by specifically focusing on immune-related proliferation markers, potentially offering higher sensitivity in predicting immunotherapy responses.

Among the identified genes, CDK1 has been well-documented as a crucial driver of cell cycle progression where its overexpression is frequently correlated with aggressive tumor growth and acts as a poor prognostic marker in pancreatic cancer (34), while AHNAK has been implicated in promoting tumor metastasis and epithelial-mesenchymal transition (EMT) process (36), lending strong biological plausibility to our risk model, suggesting that it captures the intrinsic malignant potential of the tumor.

Among the identified genes, CDK1 has been well-documented as a crucial driver of cell cycle progression. Beyond its role in tumor proliferation, elevated CDK1 activity in the tumor microenvironment may suppress T cell activation and promote immune evasion by altering cytokine secretion profiles. Similarly, AHNAK has been implicated in promoting tumor metastasis and the epithelial-mesenchymal transition (EMT) process. Its overexpression may contribute to the formation of a dense desmoplastic stroma, creating a physical barrier that restricts the infiltration of effector T cells. The inclusion of these genes lends strong biological plausibility to our risk model, suggesting it captures both the intrinsic malignant potential and the immunosuppressive stroma of the tumor.

Beyond prognosis, our findings shed new light on the complex immune evasion mechanisms in PC. NMF-based molecular subtype analysis revealed striking heterogeneity in the immune microenvironment between distinct subtypes. Specifically, Cluster 2 (associated with poor prognosis) aligned with the high-risk group identified by our model (33), which serves as a computational proxy for immune resistance. This suggests that the immune systems of these patients may struggle to effectively recognize and eliminate tumor cells due to physical or functional barriers. This aligns with the concept of the “cold” tumor phenotype often observed in PC, characterized by a dense stromal barrier, T-cell exclusion and profound immunosuppression (37). Furthermore, the upregulation of key genes like CDK1 in the high-risk group is not only linked to cell cycle dysregulation but may also participate in suppressing T-cell functions via immune checkpoint modulation or by altering the cytokine milieu (38), ultimately leading to significantly shortened overall survival. Consequently, this immunosuppressive microenvironment highlights the urgent need for strategies that can convert these “cold” tumors into “hot” ones.

Notably, a major strength of this study is the utilization of multidimensional experiments to confirm the pivotal role of IL1RN in PC, moving beyond pure bioinformatics prediction. Experimental results demonstrated significant overexpression of IL1RN in PC tissues compared to normal controls. Crucially, the knockdown of IL1RN in T cells significantly enhanced their capacity to kill tumor cells. These results provide direct evidence that IL1RN serves as a potent negative regulator of T-cell anti-tumor immunity in the pancreatic tumor microenvironment. Biologically, IL1RN encodes the interleukin-1 receptor antagonist (IL-1Ra), which competitively blocks IL-1 signaling (39). The role of IL-1 in cancer is often paradoxical; while IL-1 blockade is often explored to reduce chronic tumor-promoting inflammation (40), our data suggest that in the specific context of T-cell effector function, IL1RN-mediated suppression of IL-1 signaling appears to hinder effective anti-tumor cytotoxicity, implying that IL1RN may act similarly to an immune checkpoint. Targeting IL1RN could theoretically unleash T-cell activity, offering a promising strategy for reversing the immunosuppressive state of PC.

Furthermore, the risk model revealed a significant disparity in drug sensitivity. While high-risk patients exhibited distinct immune exclusion and resistance to immunotherapy, they showed paradoxically higher sensitivity to chemotherapy. This observation aligns with the immunosuppressive phenotype, where rapid tumor proliferation renders cells susceptible to cytotoxic agents. This suggests that low-risk patients might benefit from ICB, whereas high-risk patients are better candidates for chemotherapy or combination strategies targeting the stroma.

However, several limitations in this study must be acknowledged to provide a balanced perspective. First, the prognostic model was primarily developed using retrospective data from public databases; therefore, selection bias cannot be entirely ruled out, and large-scale, multicenter prospective clinical cohorts are required to further validate its predictive efficacy in real-world settings. Second, while algorithms like ssGSEA (41) and TIDE (33) predicted differences in immune infiltration and therapeutic response, these computational inferences are indirect and cannot fully replace the complex spatial cellular interactions within tumor tissues. Finally, although our *in vitro* experiments confirmed the immunosuppressive function of IL1RN, our validation was restricted to co-culture assays without *in vivo* animal experiments. The complex spatial cellular interactions and systemic immune responses cannot be fully recapitulated *in vitro*. Future studies utilizing *in vivo* animal models, such as Panc02 subcutaneous tumor models in immunocompetent mice, are indispensable to evaluate the dynamic impact of IL1RN on the tumor microenvironment. Furthermore, the underlying molecular pathways—such as whether it involves specific TCR signaling alterations or metabolic reprograming (19)—remain to be fully elucidated. Future studies utilizing single-cell sequencing and proteomics are warranted to provide deeper mechanistic insights (17).

In conclusion, this study provides an accurate and biologically interpretable prognostic model for PC and identifies IL1RN as a critical mediator of immune resistance, offering a robust theoretical foundation for the development of precision medicine strategies and novel immunotherapeutic interventions in PC.

## Data Availability

The datasets presented in this study can be found in online repositories. The names of the repository/repositories and accession number(s) can be found in the article/Supplementary material.

## Footnotes

1
https://portal.gdc.cancer.gov/

2
http://tide.dfci.harvard.edu/
