# Supplementary figures and images for: Prognostic value and immunological significance of t-cell proliferation regulators in pancreatic cancer: a novel predictive model
Source: Front Med (Lausanne). 2026 May 5;13:1800810. doi: 10.3389/fmed.2026.1800810 (PMC13183664; doi:10.3389/fmed.2026.1800810)

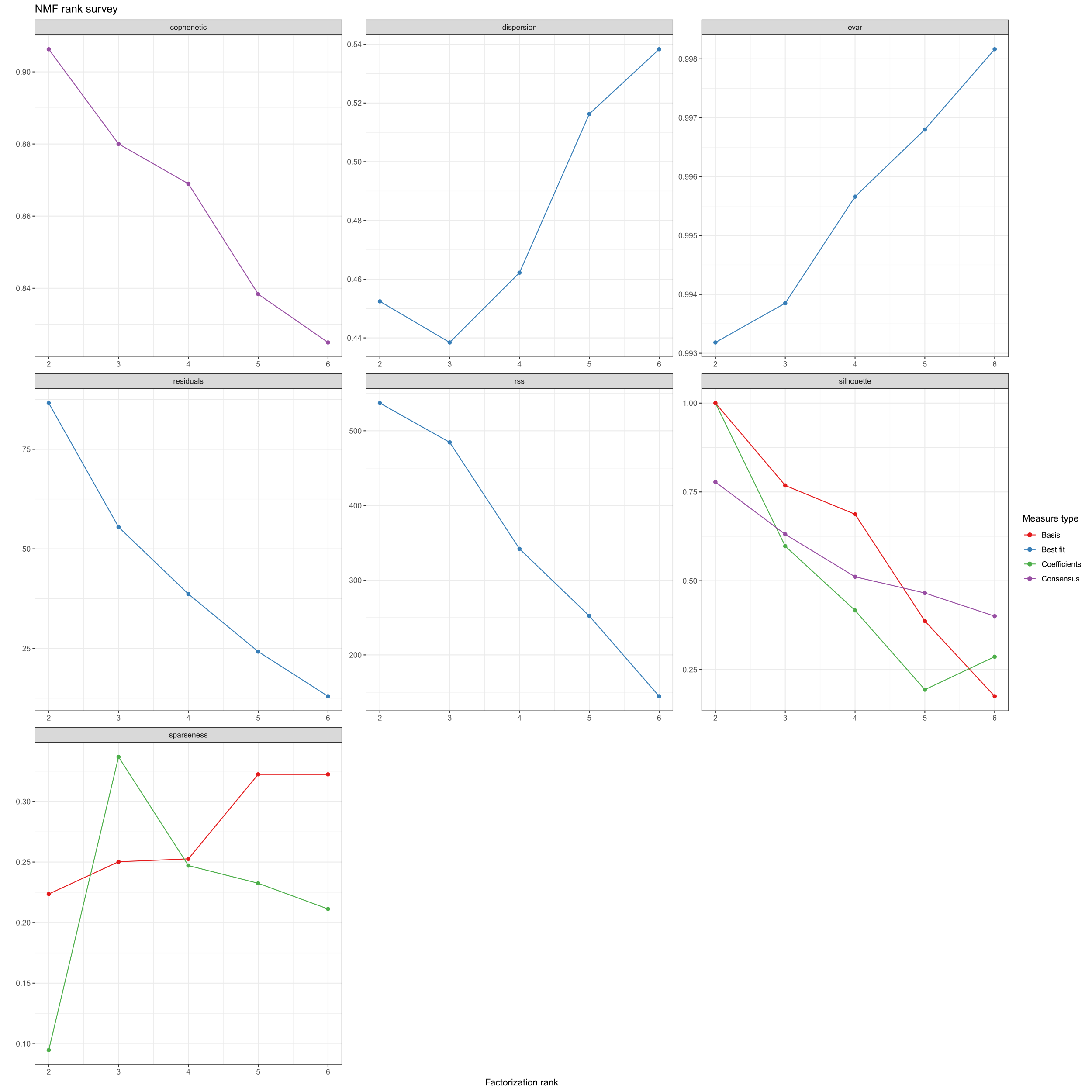

Supplement: Supplementary file 1 [file Image_1.tiff]
